# Excessive Tums Intake Can Cause Colonoscope Malfunction

**DOI:** 10.7759/cureus.3052

**Published:** 2018-07-25

**Authors:** Joshua Anderson, Ciel Harris, Amie Deutch

**Affiliations:** 1 Gastroenterology, University of Florida College of Medicine Jacksonville, Jacksonville, USA; 2 Internal Medicine, University of Florida College of Medicine-Jacksonville, Jacksonville, USA; 3 Gastroenterology, University of Florida College of Medicine-Jacksonville, Jacksonville, USA

**Keywords:** colorectal cancer screening, calcium carbonate, colonoscope malfunction

## Abstract

During a diagnostic esophagogastroduodenoscopy (EGD) and colonoscopy, a foreign material was found coating a patient’s stomach and proximal colon. Polypectomy with a hot snare and cold forceps proved unsuccessful, as the endoscope channels clogged. Thereafter, the patient confessed to taking one bottle of Tums (GlaxoSmithKline, St. Louis, Missouri, US) daily for an unknown duration. The medication was discontinued and a repeat colonoscopy showed complete resolution. The costs of repeat procedures, reduced efficacy, as well as equipment damage or refurbishment are substantial, and so providers should note that this may be the result of excessive calcium carbonate (similarly to barium) and instruct the patients to adjust intake accordingly.

## Introduction

Colonoscopy is widely accepted as the preferred method for colorectal cancer screening given its ability to identify and remove precancerous polyps [1]. Colonoscope malfunction is rare and not commonly reported. However, if encountered, a malfunction can interfere with this primary goal. Barium sulfate is an insoluble enteric contrast agent used in radiologic procedures, has been known to clog biopsy channels on endoscopes, and causes endoscope malfunction [2]. Tums is an over-the-counter antacid with calcium carbonate as its active ingredient. Here, we present a case of excessive Tums self-administration, resulting in a colonoscopy biopsy channel malfunction.

## Case presentation

**Figure 1:**
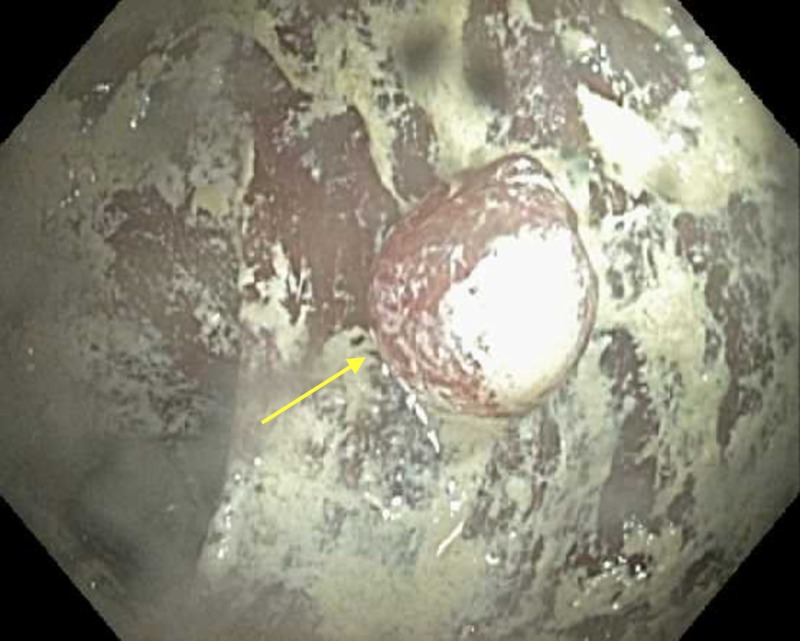
Ascending colon, single polyp. Unknown substance.

A 61-year-old African American female with a history of chronic gastroesophageal reflux disease (GERD), dyspepsia, abdominal bloating, early satiety, alternating constipation, and diarrhea was referred to the gastroenterology clinic for diagnostic esophagogastroduodenoscopy (EGD) and colonoscopy. Her physical examination and laboratory workup were unremarkable. No radiographic examinations involving oral or rectal contrast had been performed. She reported compliance with split dose 4L polyethylene glycol 3350 solution (GoLYTELY, Braintree Laboratories, Inc., MA, US) bowel preparation. She was intubated for the procedures given her medical comorbidities. During her EGD, she was noted to have a scant amount of opaque, whitish, chalky residue in her stomach, along with a hiatal hernia, and was otherwise unremarkable. The digital rectal examination performed prior to colonoscopy revealed a similar substance with a more yellowish tinge. Colonoscopy revealed this substance coating significant portions of the colonic mucosa. It was first encountered in the distal sigmoid and was present throughout the colon proximally. The substance was initially suspected to be barium sulfate; however, the patient had not previously undergone any radiographic studies using enteric contrast agents. It was unable to be cleared and interfered with the visualization of the mucosa. On withdrawal, a large pedunculated polyp was found in the ascending colon (Figure 1). The polyp was estimated to be 15 mm in size. A hot snare polypectomy was planned; however, the snare was unable to advance through the biopsy channel of the colonoscope despite several passes and the attempt was abandoned. On further withdrawal, several small polyps were seen in the sigmoid colon. Again polypectomy was attempted using cold biopsy forceps; however, was unsuccessful due to an inability to pass the forceps secondary to the clogged channel thought to be due to this substance. The colonoscopy was aborted and the patient was successfully extubated.

**Figure 2:**
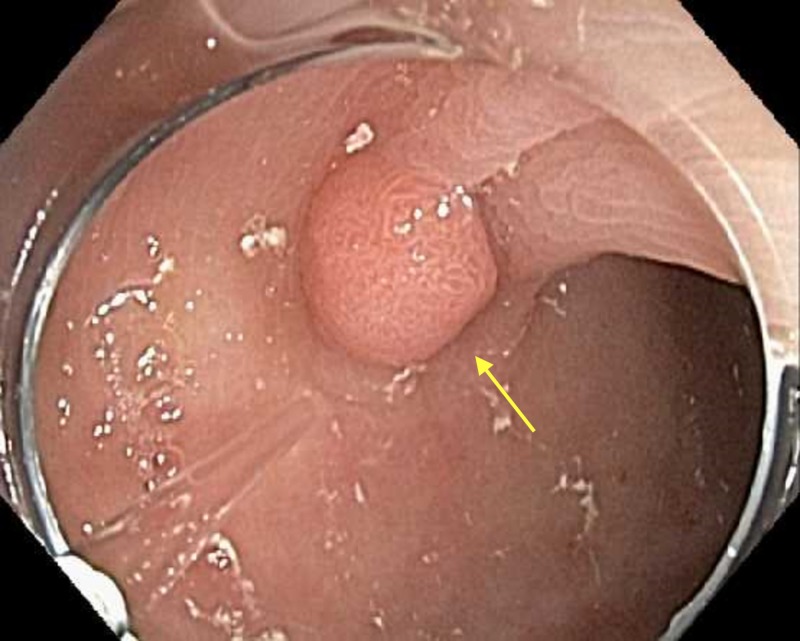
Ascending colon. Resolution of unknown substance.

At this point, she was again questioned and denied any previous studies, investigations, or enteric contrast. She did, however, admit to taking one bottle of Tums almost every day for an unspecified amount of time. The patient was instructed to discontinue her Tums medication. Repeat colonoscopy one month later showed complete resolution of the foreign substance (Figure 2), allowing for the successful polypectomy of the retained polyps. A histopathological examination revealed a 9 mm tubular adenoma in the ascending colon and several small hyperplastic polyps in the sigmoid colon. The clogged biopsy channel on the colonoscope used during the sentinel colonoscopy was easily cleared with standard cleaning practices and did not require refurbishment.

## Discussion

The evidence from this case supports the fact that excessive oral intake of calcium carbonate can impair the efficacy of colonoscopy and cause endoscope malfunction. A review of the literature indicates that the finding of a malfunctioning colonoscope secondary to the excessive patient intake of calcium carbonate has not previously been reported. The lack of abnormal material on repeat colonoscopy after the patient stopped taking calcium carbonate along with her denial of enteric contrast prior to her original procedure strongly suggest that the excessive intake of Tums was the culprit. We believe that the excessive intake was likely contributing to her alternating bowel habits through fluctuating levels of serum calcium and osmotic effects on the colon. Calcium carbonate is primarily excreted through the colon (75%) as unabsorbed calcium.

Endoscopic equipment and procedures are expensive. The four leading endoscopic equipment manufacturers are Fujinon Inc (Valhalla, NY, US), Olympus America (Center Valley, PA, US), Pentax Precision Instruments Inc (Orangeburg, NY, US), and Welch-Allyn Inc (Skaneateles Falls, NY, US) [3]. The price of a colonoscope can range from $2900-22000 (refurbished) with currently 13 colonoscope devices registered with the United States Food and Drug Administration (FDA) [4]. Without insurance, the average patient should expect to pay $2500-$3000 for a screening colonoscopy [5]. Considerable savings are available through various insurance companies. The costs of repeat procedures are not unsubstantial either, including lost productivity, equipment, labor, etc. [6-8]. In this case, the patient was exposed to the inherent risks of colonoscopy without the benefit of polypectomy and was required to repeat the procedure.

We contacted Olympus Tokyo (Tokyo, Japan) through our liaison and we were informed that the endoscope material is not affected by barium, through damage, or chemical changes. However, barium is a water-insoluble substance and could solidify in and on the endoscope if not immediately removed in a timely manner following the procedure. This could lead to a partial or full channel blockage and may also pose an infection control risk. Current recommendations have been made to suction and aspirate copious volumes of water through every channel immediately following the procedure and then immediately reprocess the endoscope. Providers should be aware that a patient's excessive calcium carbonate intake may result in the impaired efficacy of colonoscopy and endoscope malfunction, and instruct patients to adjust the intake accordingly.

## Conclusions

The case demonstrates that excessive oral intake of calcium carbonate can disrupt the ability to successfully complete colonoscopy and may lead to endoscope malfunction. Patients suspected of consuming excessive amounts of calcium carbonate should discontinue use several days prior to colonoscopy to minimize the risk of needing repeat procedures.
